# Effect of synergistic interaction between abnormal adiposity-related metabolism and prediabetes on microalbuminuria in the general population

**DOI:** 10.1371/journal.pone.0180924

**Published:** 2017-07-17

**Authors:** Jong Wook Choi, Il Hwan Oh, Chang Hwa Lee, Joon-Sung Park

**Affiliations:** Department of Internal Medicine, Hanyang University College of Medicine, Seoul, Korea; The University of Tokyo, JAPAN

## Abstract

Central obesity and related metabolic components are important risks for microalbuminuria. To describe the effects of interactions between central obesity and related metabolic components on microalbuminuria, we conducted a nation-wide, population-based interaction analysis using cardio-metabolic index (CMI) as a candidate indicator of central obesity and related abnormal lipid metabolism. We recruited native Koreans aged 20 years or older with no medical illness. A total of 5398 participants were divided into quintiles according to CMI with sex as a covariate factor. Participants in the highest CMI quintile had elevated blood pressure (BP), increased glycemic exposure, poor lipid profile, and increased urine albumin-to-creatinine ratio compared to other lower quintiles. Multiple logistic regression models adjusted for age, sex, systolic BP, and diastolic BP showed that CMI had an independent association with increased glycemic exposure and increased urine albumin-to-creatinine ratio. Our interaction analysis revealed a significant interaction between the highest CMI quintile and prediabetes with an increased risk of microalbuminuria (adjusted RERI = 0.473, 95% *CI* = 0.464–0.482; adjusted AP = 0.276, 95% *CI* = 0.156–0.395; adjusted SI = 2.952, 95% *CI* = 1.234–4.670). Our findings suggest a significant association between central obesity-related abnormal lipid metabolism and prediabetes, and their interaction may exert a synergistic effect on renal vascular endothelial dysfunction even before the appearance of full-blown diabetes mellitus. To confirm these findings, large population-based prospective studies are needed.

## Introduction

Central obesity and related abnormal lipid metabolism are main components of metabolic syndrome, which is a major public health and economic problem on the global scale [1]. Accumulating evidence indicates that the copious release of free fatty acids and various pro-inflammatory cytokines/adipokines from dysfunctional adipose tissue plays a central role in the development of low-grade systemic inflammation and metabolic complications in patients with visceral obesity [2]. Accurate measurement of central obesity is critical to assess the severity of its metabolic disturbances and to prevent irreversible metabolic disorders. Recently, the cardio-metabolic index (CMI), simply calculated as the product of waist-to-height ratio (WHtR) and triglycerides (TG) to high-density lipoprotein (HDL) cholesterol ratio, was introduced as a possible clinical indicator reflecting central obesity and related abnormal lipid metabolism; CMI has been shown to describe the risk of diabetes in the general population and also had an association with progression of atherosclerosis in patients with peripheral arterial disease [3, 4], suggesting that CMI may be a useful marker for endovascular damage. However, little is known about its role in estimating the negative effects of central obesity-related abnormal lipid metabolism on kidney function.

Prediabetes refers to a plasma glucose level above the normal range but not high enough to meet the diagnostic criteria for diabetes [5]. Previous epidemiological studies have demonstrated that increased glycemic exposure is independently associated with glomerular hyperfiltration, vascular endothelial dysfunction, and hyperglycemia-related metabolic disturbance, and these conditions are strongly linked to the development of chronic kidney disease as well as cardiovascular disease [6–8]. Recently, some authors have argued that various forms of vascular complications associated with diabetes may be prominent in subjects with prediabetes [9], suggesting that mild hyperglycemia itself may be a leading cause of vascular endothelial dysfunction, independent of central obesity and related abnormal lipid metabolism.

Microalbuminuria indicates a small increase in the leakage of albumin into urine, but it may implicate the presence of glomerular filtration barrier dysfunction, a significant feature of microvascular complication in patients with diabetes [10]. It is widely accepted that the development of vascular endothelial damage is independently associated with central obesity-related abnormal lipid metabolism as well as hyperglycemia-related metabolic disturbances, and there is close link between them [11, 12]. However, there is little clinical evidence of their interaction, especially in the general population. Thus, to directly address this relationship, we conducted an interaction analysis using CMI as a candidate indicator of abnormal central obesity-related lipid metabolism.

## Subjects and methods

### Study population

All data were collected from public-use data sets from the Korean National Health and Nutrition Examination Survey (KNHANES) conducted by the Korea Centers for Disease Control and Prevention (KCDC) among non-institutionalized Korean civilians between 2013 and 2014. All participants were volunteers, and all provided written informed consent before enrollment in the study. Their records, except for the date of the survey, were anonymized prior to analysis. The study protocol was approved by the Institutional Review Board (IRB) of the KCDC (IRB: 2013-07CON-03-4C, 2013-12EXP-03-5C).

A total of 15568 individuals participated in the KHANES 2013–2014. Individuals were excluded from the present analysis for any of the following reasons: (1) incomplete anthropometric or laboratory data, (2) < 20 years of age, (3) pregnancy, (4) a history of medical problems, such as diabetes, hypertension, cardiovascular diseases, and malignancies, (5) estimated glomerular filtration rate (eGFR) < 60 mL/min/1.73 m^2^, or (6) urine albumin-to-creatinine ratio (UACR) > 300 mg/g creatinine. The final 5398 participants were divided into quintiles according to CMI and were categorized by sex (S1 Fig).

### Anthropometric and clinical measurements

Anthropometric measurements were performed by well-trained examiners. Participants wore a lightweight gown or underwear. Height (Ht) was measured to the nearest 0.1 cm using a portable stadiometer (Seriter, Bismarck, ND). Weight was measured to the nearest 0.1 kg on a calibrated balance-beam scale (Giant-150N; Hana, Seoul, Korea). Waist circumference (WC) was measured using a flexible tape at the narrowest point between the lowest border of the rib cage and the uppermost lateral border of the iliac crest at the end of normal expiration. BMI was calculated as weight in kilograms divided by square of the height in meters. WHtR was calculated as WC divided by height.

Blood pressure (BP) was measured three times using a mercury sphygmomanometer (Baumanometer; Baum, Copiague, NY) while subjects were in a sitting position following a 5-minute rest period. The average values of the three recorded systolic and diastolic BPs were used in the analysis.

### Laboratory tests

Venous blood samples were collected after 8 h overnight fasting. Fasting plasma concentrations of glucose, low-density lipoprotein cholesterol, HDL cholesterol, and TG were determined using a Hitachi Automatic Analyzer 7600 (Hitachi, Tokyo, Japan). Glycated hemoglobin (HbA1c) levels were determined using high-performance liquid chromatography with an automated HLC-723G7 analyzer (Tosoh Corporation, Tokyo, Japan). Serum creatinine levels were measured colorimetrically (Hitachi Automatic Analyzer 7600), and eGFR was calculated using the Chronic Kidney Disease Epidemiology Collaboration equation (CKD-EPI) [13]. To obtain the UACR, urinary albumin was measured in spot urine using the immunoturbidimetric method, and urinary creatinine was measured using the colorimetric method.

### Definitions

According to the 2016 American Diabetes Association standards of medical care [14], prediabetes was defined as a fasting glucose between 100 and 125 mg/dl or HbA1c in the range 5.7 to 6.4% without the use of hypoglycemic medications. According to the KDIGO 2012 Clinical Practice Guidelines [15], participants with microalbuminuria were defined as those with a UACR between 30 and 300 mg/g creatinine, and participants with mild decline of kidney function were defined as those with an eGFR between 60 and 89 mL/min/1.73 m^2^.

### Statistical analysis

All data, including socio-demographic information, medical conditions, anthropometric and clinical measurements, and laboratory results, were presented as mean ± SE or frequency (and proportion). The normality of the distribution of parameters was analyzed by the Kolmogorov-Smirnov test. Data were analyzed using sampling weights to account for multistage and stratified sampling. The generalized linear model was used to compare quantitative variables, and the Chi-square test was used to compare proportions for categorical variables. Linear regression analysis was used to assess the relationships between potential predictor variables and CMI. Odds ratios (ORs) with 95% confidence intervals (*CI*s) were calculated in multiple logistic regression models according to the presence of microalbuminuria (case vs. control). Age and sex were initially adjusted as covariates in both linear regression and multiple logistic regression models. Further adjustment was made for the covariates that were significant in the first analyses. Other clinical and laboratory variables were evaluated as predictors using regression analyses.

Receiver operating characteristic (ROC) curves were used to compare the predictive capacity of CMI and anthropometric indices of central obesity on microalbuminuria. The nonparametric method of Delong was used to compare the areas under the ROC curves (AUCs) [16].

A biological interactive effect between candidate risk factors on dependent variables was evaluated on both a multiplicative scale and additive scales using logistic regression analysis [17]. The interaction analysis based on the multiplicative scale was performed by comparing participants in the highest CMI quintile, participants with prediabetes, or participants with both to participants in the first 4 quintiles with normal glycemic exposure. The interaction based on the additive scale was evaluated by 3 indices: RERI, the relative excess risk due to the interaction; AP, the attributable proportion due to the interaction; and SI, the additive interaction index of synergy [18]. RERI is the excess risk due to the interaction relative to the risk without exposure. AP refers to the attributable proportion of disease that is due to the interaction among individuals with both exposures. SI is the excess risk from both exposures when there is an additive interaction, relative to the risk from both exposures without interaction. If the 95% *CI* of RERI and AP included 0, and the 95% *CI* of S contained 1, it was interpreted that there was no additive interaction between candidate variables.

A two-tailed P < 0.01 was considered statistically significant. Statistical Analysis Software, version 9.4 (SAS Institute Inc., Cary, NC, USA), was used for all analyses.

## Results

### Baseline characteristics

The participants (n = 5398) comprised 2186 men and 3212 women with a mean age of 39.4 ± 0.3 years. They were divided into quintiles according to CMI and were categorized according to sex. Participants in the highest quintile were older and more hypertensive than those in the other quintiles, and they were more likely to have increased glycemic exposure, worse lipid profiles, and poor kidney function. There were no differences in other baseline characteristics across CMI quintiles (Table 1).

**Table 1:** General characteristics of the study cohort grouped according to cardio-metabolic index (CMI).

	Quintile 1	Quintile 2	Quintile 3	Quintile 4	Quintile 5	
CMI in males	≥ 0.07, ≤ 0.64	> 0.64, ≤ 0.98	> 0.98, ≤ 1.46	> 1.46, ≤ 2.29	> 2.29, ≤ 23.88	
CMI in females	≥ 0.07, ≤ 0.40	> 0.40, ≤ 0.58	> 0.58, ≤ 0.85	> 0.85, ≤ 1.34	> 1.34, ≤ 14.21	
Variables	(n = 1080)	(n = 1078)	(n = 1081)	(n = 1079)	(n = 1080)	P
Age (years)	35.2 ± 0.4	38.8 ± 0.4	40.7 ± 0.5	43.6 ± 0.5	44.6 ± 0.5	<0.0001
Sex (% male)	438 (41)	436 (40)	438 (41)	436 (40)	438 (41)	0.9726
Systolic BP (mmHg)	107.4 ± 0.4	108.5 ± 0.4	110.2 ± 0.4	111.6 ± 0.4	114.4 ± 0.4	<0.0001
Diastolic BP (mmHg)	70.4 ± 0.3	71.6 ± 0.3	72.8 ± 0.3	73.8 ± 0.3	75.6 ± 0.3	<0.0001
Body mass index (kg/m^2^)	21.2 ± 0.1	22.3 ± 0.1	23.2 ± 0.1	24.2 ± 0.1	25.5 ± 0.1	<0.0001
Waist circumference (cm)	72.5 ± 0.3	76.3 ± 0.3	78.8 ± 0.3	81.9 ± 0.3	85.4 ± 0.3	<0.0001
eGFR* (mL/min/1.73 m^2^)	105.5 ± 0.5	102.4 ± 0.4	100.7 ± 0.5	99.3 ± 0.5	99.6 ± 0.5	0.0002
Hemoglobin (g/dL)	14.09 ± 0.05	14.09 ± 0.06	14.28 ± 0.06	14.32 ± 0.05	14.49 ± 0.05	<0.0001
Fasting glucose (mg/dL)	89.3 ± 0.3	91.5 ± 0.3	92.9 ± 0.3	94.2 ± 0.3	96.1 ± 0.4	<0.0001
Hemoglobin A1c (%)	5.45 ± 0.01	5.50 ± 0.01	5.53 ± 0.01	5.60 ± 0.01	5.66 ± 0.01	<0.0001
Triglycerides (mg/dL)	53.2 ± 0.5	78.4 ± 0.7	103.3 ± 1.0	137.9 ± 1.4	261.0 ± 5.4	<0.0001
HDL-cholesterol (mg/dL)	66.4 ± 0.4	57.4 ± 0.4	53.0 ± 0.3	48.6 ± 0.3	42.8 ± 0.3	<0.0001
LDL-cholesterol (mg/dL)	98.8 ± 0.9	107.4 ± 1.0	108.9 ± 6.8	124.0 ± 4.2	118.4 ± 1.6	<0.0001
25-Vitamin D (ng/mL)	16.1 ± 0.4	16.0 ± 0.3	16.3 ± 0.3	16.2 ± 0.4	15.8 ± 0.4	0.8194
Log-UACR (log mg/g Cr)	1.30 ± 0.04	1.13 ± 0.04	1.26 ± 0.04	1.25 ± 0.04	1.44 ± 0.05	0.0093

Results are expressed as mean ± SD or frequency (and proportion).

BP, blood pressure; eGFR, estimated glomerular filtration rate; HDL, high-density lipoprotein; LDL, low-density lipoprotein; Log-UACR, log-transformed urine albumin/creatinine ratio; Cr, creatinine.

*estimated using the Chronic Kidney Disease Epidemiology Collaboration (CKD-EPI) equation.

### The relationships between CMI, kidney function, and other related risk factors

We performed linear regression analysis with age and sex as covariates to assess the relationships between CMI and other baseline characteristics related to kidney function. As shown in S1 Table, we found that CMI had a significant relationship with important components of metabolic syndrome including systolic BP, diastolic BP, glycemic exposure, dyslipidemia, and central obesity.

### Association of CMI with microalbuminuria

We performed multiple logistic regression models to quantify the associations between CMI and other candidate predictors for kidney disease. First, we found that there was a significant association between CMI and prediabetes in this population (adjusted OR = 1.080, 95% *CI* = 1.021–1.142, Table 2). Furthermore, our logistic models demonstrated that CMI also had an independent association with microalbuminuria (crude OR = 1.074, 95% *CI* = 1.006–1.145) regardless of age and sex, and further adjustment for age, sex, systolic BP, and diastolic BP did not attenuate this association (adjusted OR = 1.075, 95% *CI* = 1.001–1.154, Table 3). However, we could not observe an independent association between CMI and mild declined kidney function (S2 Table).

**Table 2:** Multivariable logistic regression for prediabetes*.

	Crude		Model I		Model II		Model III	
Variable	OR	95% *CI*	OR	95% *CI*	OR	95% *CI*	OR	95% *CI*
Age (years)	1.054	1.049–1.060						
Female (vs. male)	0.681	0.601–0.772						
Smoker (vs. non-smoker)	1.263	1.081–1.475	1.159	0.964–1.393				
Systolic BP (mmHg)	1.033	1.026–1.039	1.015	1.009–1.022				
Diastolic BP (mmHg)	1.035	1.027–1.044	1.021	1.011–1.030				
Body mass index (kg/m^2^)	1.139	1.117–1.162	1.120	1.098–1.142	1.111	1.089–1.134		
Waist circumference (cm)	1.056	1.048–1.063	1.045	1.037–1.053	1.402	1.034–1.050		
eGFR (mL/min/1.73 m^2^)	0.965	0.961–0.969	0.996	0.990–1.002				
Hemoglobin (g/dL)	1.018	0.978–1.060						
Fasting glucose (mg/dL)	1.193	1.178–1.208	1.177	1.161–1.193	1.177	1.161–1.196	1.173	1.156–1.189
Hemoglobin A1c (%)	999.9	999.9–999.9	999.9	999.9–999.9	999.9	999.9–999.9	999.9	999.9–999.9
Triglycerides (mg/dL)	1.003	1.003–1.004	1.002	1.002–1.003	1.002	1.001–1.003	1.001	1.000–1.002
HDL-cholesterol (mg/dL)	0.976	0.971.981	0.984	0.978–0.990	0.985	0.979–0.990	0.993	0.987–0.999
LDL-cholesterol (mg/dL)	1.005	1.000–1.011	1.004	0.998–1.009				
CMI	1.312	1.221–1.411	1.194	1.121–1.273	1.174	1.105–1.248	1.080	1.021–1.142
25-Vitamin D (ng/mL)	1.034	1.018–1.049	1.002	0.985–1.019				
UACR (mg/g creatinine)	1.006	1.001–1.010	1.004	1.000–1.008	1.003	0.999–1.007		

*defined as fasting glucose between 100 and 125 or hemoglobin A1c between 5.7 and 6.4%.

Model I, adjusted for age and sex

Model II, adjusted for age, sex, systolic BP, and diastolic BP.

Model III, adjusted for age, sex, systolic BP, diastolic BP, body mass index, and waist circumference.

OR, odd ratio; *CI*, confidence interval; Log-UACR, log-transformed urine albumin-to-creatinine ratio.

**Table 3:** Multivariable logistic regression for microalbuminuria*.

	Crude		Model I		Model II	
Variable	OR	95% *CI*	OR	95% *CI*	OR	95% *CI*
Age (years)	1.028	1.018–1.038				
Female (vs. male)	1.729	1.223–2.442				
Smoker (vs. non-smoker)	0.692	0.426–1.125				
Systolic BP (mmHg)	1.031	1.013–1.050	1.028	1.010–1.047		
Diastolic BP (mmHg)	1.024	1.001–1.047	1.030	1.007–1.054		
Body mass index (kg/m^2^)	1.063	0.999–1.132				
Waist circumference (cm)	1.018	0.996–1.039				
eGFR (mL/min/1.73 m^2^)	0.996	0.984–1.007				
Hemoglobin (g/dL)	0.940	0.835–1.059				
Fasting glucose (mg/dL)	1.036	1.014–1.058	1.032	1.010–1.055	1.027	1.005–1.050
Hemoglobin A1c (%)	1.474	0.872–2.492				
Triglycerides (mg/dL)	1.001	1.000–1.002	1.002	1.000–1.003	1.001	1.000–1.002
HDL-cholesterol (mg/dL)	0.990	0.977–1.004				
LDL-cholesterol (mg/dL)	1.011	0.999–1.023				
CMI	1.074	1.006–1.145	1.097	1.026–1.173	1.075	1.001–1.154
25-Vitamin D (ng/mL)	1.015	0.979–1.052				

*defined as UACR (mg/g creatinine) between 30 and 300.

Model I, adjusted for age and sex.

Model II, adjusted for age, sex, systolic BP, and diastolic BP.

OR, odd ratio; *CI*, confidence interval.

**Fig 1:**
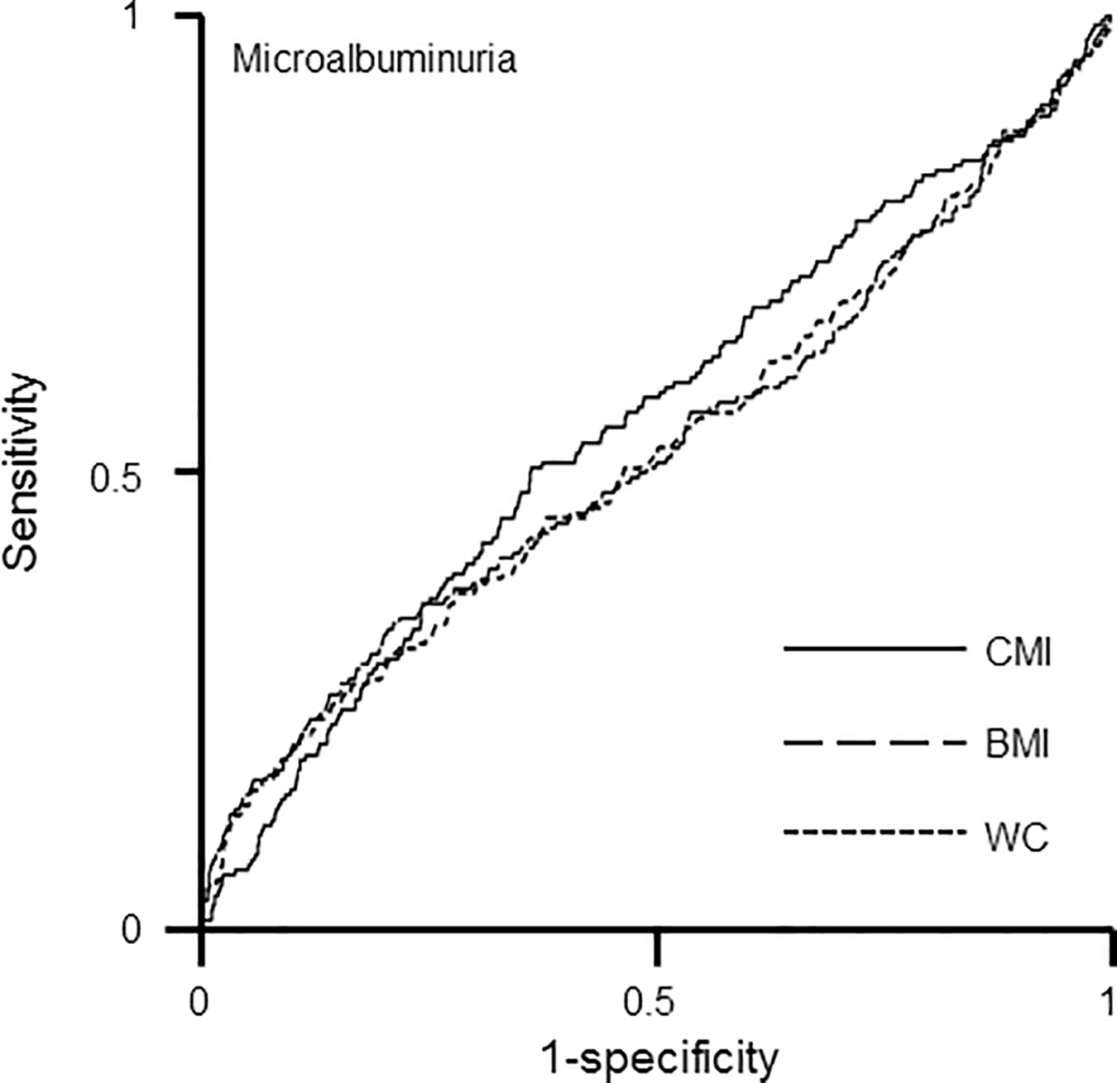
Receiver-operating characteristic (ROC) curves representing the prediction capacity of risk for (B) microalbuminuria. Compared with WC (AUC* = 0.5199, 95% CI* = 0.4716–0.5681), CMI (AUC* = 0.5523, 95% CI* = 0.5069–0.5977, P* = 0.0662) and BMI (AUC* = 0.5223, 95% CI* = 0.4732–0.5713, P* = 0.6369) have better precision in predicting microalbuminuria, but the difference was not statistically significant. BMI; body mass index; WC, waist circumference; AUC, area under the curve; *CI*, confidence interval. *calculated by logistic regression analysis using age, sex, systolic BP, and diastolic BP as covariates.

To obtain predictive values between CMI and conventional anthropometric indices of central obesity for microalbuminuria, we created receiver operating characteristic (ROC) curves by performing a logistic regression model with age, sex, systolic BP, and diastolic BP as covariates and compared the AUCs (Fig 1). Compared with WC (AUC = 0.5199, 95% *CI* = 0.4716–0.5681), CMI (AUC = 0.5523, 95% *CI* = 0.5069–0.5977, P = 0.0622) and BMI (AUC = 0.5223, 95% *CI* = 0.4732–0.5713, P = 0.6369) had higher predictive values for microalbuminuria, but the difference between CMI and WC was marginal.

### Biological interaction analysis between central obesity-related abnormal lipid metabolism and increased glycemic exposure on microalbuminuria

To examine the interactive effects between central obesity-related abnormal lipid metabolism and increased glycemic exposure on kidney dysfunction, we performed multiplicative and additive interaction analyses. As shown in Table 4, compared with participants in the first 4 quintiles without prediabetes, participants with just prediabetes or those in the highest quintile had no significant risk for microalbuminuria after adjustment for age, sex, systolic BP, and diastolic BP, and the adjusted ORs were 1.324 (95% *CI* = 0.716–2.449; first 4 quintiles only) and 1.001 (95% *CI* = 0.665–1.525; pre-diabetes only). In contrast, participants in the highest quintile with prediabetes had a significantly increased risk for microalbuminuria (crude OR = 2.676, 95% *CI* = 1.621–4.416), and adjustment for age, sex, systolic BP, and diastolic BP did not affect this multiplicative interaction (adjusted OR = 2.008, 95% *CI* = 1.233–3.271).

**Table 4:** Interactive effect analysis of CMI quintile and prediabetes on microalbuminuria.

Categories		Unadjusted		Adjusted*	
Prediabetes	CMI quintile	OR	95% *CI*	OR	95% *CI*
(-)	1–4	1 (reference)		1 (reference)	
(-)	5	1.601	0.882–2.903	1.324	0.716–2.449
(+)	1–4	1.147	0.756–1.741	1.001	0.665–1.525
(+)	5	2.676	1.621–4.416	2.008	1.233–3.271

* adjusted for age, sex, systolic BP, and diastolic BP.

In additive interaction analysis (Table 5), we found that there was increased risk of microalbuminuria in participants in the highest quintile with prediabetes, attributed to the interaction between them, even after adjustment for age, sex, systolic BP, and diastolic BP (adjusted AP = 27.6%, 95% *CI* = 0.156–0.395). Furthermore, the interaction index for synergy was 2.952 (95% *CI* = 1.234–4.670), suggesting that there was a synergistic interaction between central obesity-related abnormal lipid metabolism and increased glycemic exposure in the risk of microalbuminuria, even before the appearance of overt hypertension and diabetes.

**Table 5:** Index of additive biological interactive effect of CMI quintile and prediabetes on microalbuminuria.

	Unadjusted		Adjusted*		
Measure	Estimate	95% *CI*	Estimate	95% *CI*
RERI	0.800	0.789–0.811	0.473	0.464–0.482
AP	0.316	0.280–0.423	0.276	0.156–0.395
SI	2.090	0.644–3.536	2.952	1.234–4.670

* adjusted for age, sex, systolic BP, and diastolic BP.

If there was no biological interaction, the 95% *CI* of RERI and AP included 0, and the 95% *CI* of SI contained 1.

RERI, relative excess risk because of the interaction; AP, attributable proportion of the interaction; SI, additive interaction index of synergy.

## Discussion

This study provides comprehensive information on the performance of CMI as a surrogate indicator for central obesity-related abnormal lipid metabolism with respect to kidney function. We demonstrated that there is a positive synergistic interactive relationship between CMI and mild hyperglycemia on the risk of microalbuminuria in the general population. These results indicate that central obesity-related abnormal lipid metabolism and its metabolic interaction with glycemic exposure may be one of the leading causes of vascular endothelial dysfunction and kidney disease, even before the diagnosis of diabetes and hypertension.

In this study, we used CMI as a possible indicator of central obesity-related abnormal lipid metabolism. Our logistic regression models and ROC curve analysis revealed that CMI was independently associated with the risk of microalbuminuria. Moreover, CMI was more precise than conventional anthropometric indices in estimating the negative effects of central obesity-related abnormal lipid metabolism on kidney dysfunction. Currently, BMI and WC are widely used anthropometric indices in estimating body fat mass, but they have some limitations. BMI for measurement of obesity is not accurate to assess central adiposity because BMI is likely to overestimate obesity in adults with edema, a large amount of muscle mass, and/or peripheral fat distribution [19]. On the other hand, WC is accepted as a key metric of central obesity that can anticipate the risk of diabetes, hypertension, and cardiovascular disease in the general population [20]. However, enlarged WC could be due to abdominal subcutaneous or visceral adipose depots [21]. Furthermore, neither BMI nor WC can fully reflect abnormal lipid profiles. Thus, there is need for a more accurate clinical indicator to evaluate the systemic effect of metabolic disturbance, and CMI may be this indicator, based on the following reasons. WHtR may be better than WC or BMI as an obesity indicator for prediction of metabolic effects on the body, including the kidney [22–25], and the TG/HDL cholesterol ratio may be better than other lipid-related ratios at predicting kidney function [26]. Furthermore, recent studies have demonstrated that obesity-related kidney disease is more related to circulating TG level and renal deposition of lipids than to body weight itself [27]. These findings suggest that CMI could be a more sensitive indicator of metabolic disturbance in kidney disease.

Our results demonstrated that CMI was strongly related to increased glycemic exposure and poor lipid profiles and was independently associated with microalbuminuria, an important clinical indicator of vascular endothelial dysfunction. Non-specific inflammatory responses are accepted as a key mechanism involved in central obesity-related target organ manifestations [28]. Previous experimental research has demonstrated that various pro-inflammatory cytokines and hormones secreted from dysregulated adipocytes and macrophages within visceral fat may lead to low-grade systemic inflammation, insulin resistance, dyslipidemia, and/or increased synthesis of vasoactive and fibrogenic substances, and their complicated interactions may have a negative influence on vascular endothelial cells and cause impairment of kidney function [29–33]. However, there is limited clinical evidence of these pathologic situations. Our study adds clinical evidence of the pathologic effect of central obesity on the vascular endothelium in company with abnormal lipid profiles and increased glycemic exposure.

Microalbuminuria is the end result of complex interactions between endocrine, metabolic, and hemodynamic factors [34]. Previous epidemiologic studies have demonstrated that, compared with healthy subjects, patients with prediabetes had increased UACR, which was closely related with progression to type 2 diabetes [35, 36]. Recently, some authors also argued that central obesity and related abnormal lipid metabolism were significantly associated with increased urinary excretion of albumin, regardless of glycemic status [37–40]. However, epidemiologic evidence of the interaction between central obesity-related abnormal lipid metabolism and dysglycemia on the development of microalbuminuria is still very limited. Our results indicate that the additive synergistic interaction between CMI and prediabetes may exert a negative effect on vascular endothelial function in the kidney.

However, we could not observe an independent association between CMI and mild decline in kidney function. This is inconsistent with other reports showing that there was a strong relationship between increased anthropometric indices and progression of kidney disease [41]. A possible explanation for this discrepancy is that this study was limited by its cross-sectional study design, which made it difficult to efficiently demonstrate the long-term harmful effects of metabolic disturbances on the changes in kidney function. Another possible explanation is that CMI may be a more reliable indicator for negative consequences of central obesity-related abnormal lipid metabolism on vascular endothelial function rather than irreversible progression to renal fibrogenesis. Thus, further studies are needed to resolve these issues.

There were several limitations to our study. First, because a very small number of participants had UACR > 300 mg/g creatinine, we could not include these participants in the study. This limitation made it impossible to investigate a relationship between metabolic disturbance and overt proteinuria. Second, the development of vascular endothelial dysfunction may have been influenced by a wide variety of factors. Because of the limitations of the study design, we could not adjust for many factors other than age, sex, and BP. Third, because of the self-reporting of medical history, medication, and use of tobacco and alcohol, a social-desirability bias cannot be ruled out. These biases might have been responsible for the results and conclusions that conflict with previous research. Finally, participants may have forgotten to report relevant details.

The results of the present study indicate that CMI could be valuable for predicting renal manifestations associated with metabolic disturbances. Furthermore, a synergistic interaction between central obesity-related abnormal lipid metabolism and prediabetes may be closely related to increased risk of microalbuminuria. A large population-based prospective epidemiologic study is needed to confirm these findings.

## Supporting information

S1 Fig Flow chart of the study group enrollment process. KHANES, The Korean National Health and Nutritional Examination Survey; Q, cardio-metabolic index (CMI) quintile.
(TIF)

S1 Table Linear regression for CMI. Model I, adjusted for age and sex.
(DOCX)

S2 Table Multivariable logistic regression for early decreased kidney function. *defined as eGFR between 60 and 89 mL/min/1.73 m^2^. Model I, adjusted for age and sex.
(DOCX)
