# Supplementary figures and images for: Effect of synergistic interaction between abnormal adiposity-related metabolism and prediabetes on microalbuminuria in the general population
Source: PLoS One. 2017 Jul 17;12(7):e0180924. doi: 10.1371/journal.pone.0180924 (PMC5513435; doi:10.1371/journal.pone.0180924)

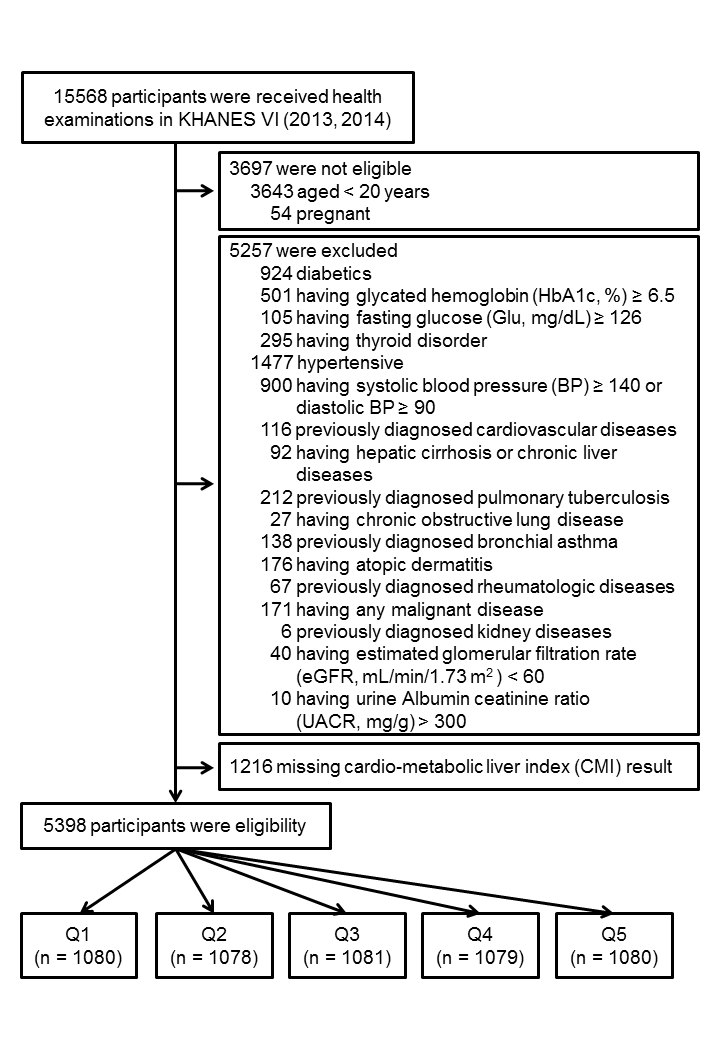

Supplement: S1 Fig — KHANES, The Korean National Health and Nutritional Examination Survey; Q, cardio-metabolic index (CMI) quintile. (TIF) [file pone.0180924.s001.TIF]
