# Supplementary material for: Effect of synergistic interaction between abnormal adiposity-related metabolism and prediabetes on microalbuminuria in the general population
Source: PLoS One. 2017 Jul 17;12(7):e0180924. doi: 10.1371/journal.pone.0180924 (PMC5513435; doi:10.1371/journal.pone.0180924)
Supplement: S1 Table — Model I, adjusted for age and sex. (DOCX) [file pone.0180924.s002.docx]

S1 Table. Linear regression for CMI

|  | Crude | |  | Model I | | |
| --- | --- | --- | --- | --- | --- | --- |
| Variable | Slope | P |  | | Slope | P |
| Age (years) | 0.0127 | <0.0001 |  | |  |  |
| Female (vs. male) | -0.7128 | <0.0001 |  | |  |  |
| Systolic BP (mmHg) | 0.0289 | <0.0001 |  | | 0.0183 | <0.0001 |
| Diastolic BP (mmHg) | 0.0377 | <0.0001 |  | | 0.0250 | <0.0001 |
| Body mass index (kg/m^2^) | 0.1771 | <0.0001 |  | | 0.1578 | <0.0001 |
| Waist circumference (cm) | 0.0699 | <0.0001 |  | | 0.0634 | <0.0001 |
| eGFR (mL/min/1.73 m^2^) | -0.0076 | <0.0001 |  | | -0.0096 | 0.0004 |
| Hemoglobin (g/dL) | 0.2182 | <0.0001 |  | | 0.1384 | <0.0001 |
| Fasting glucose (mg/dL) | 0.0377 | <0.0001 |  | | 0.0294 | <0.0001 |
| Hemoglobin A1c (%) | 0.7641 | <0.0001 |  | | 0.5654 | <0.0001 |
| Triglycerides (mg/dL) | 0.0142 | <0.0001 |  | | 0.0142 | <0.0001 |
| HDL-cholesterol (mg/dL) | -0.0624 | <0.0001 |  | | -0.0580 | <0.0001 |
| LDL-cholesterol (mg/dL) | 0.0225 | <0.0001 |  | | 0.0222 | <0.0001 |
| 25-Vitamin D (ng/mL) | -0.0044 | 0.4588 |  | |  |  |
| Log-UACR (log mg/g Cr) | 0.0041 | 0.0294 |  | | 0.0701 | 0.0061 |

Model I, adjusted for age and sex
