# Supplementary material for: Effect of synergistic interaction between abnormal adiposity-related metabolism and prediabetes on microalbuminuria in the general population
Source: PLoS One. 2017 Jul 17;12(7):e0180924. doi: 10.1371/journal.pone.0180924 (PMC5513435; doi:10.1371/journal.pone.0180924)
Supplement: S2 Table — *defined as eGFR between 60 and 89 mL/min/1.73 m2. Model I, adjusted for age and sex. (DOCX) [file pone.0180924.s003.docx]

S2 Table. Multivariable logistic regression for early decreased kidney function*****

|  | Crude | |  | Model I | |
| --- | --- | --- | --- | --- | --- |
| Variable | OR | 95% *CI* |  | OR | 95% *CI* |
| Age (years) | 1.082 | 1.075-1.089 |  |  |  |
| Female (vs. male) | 0.627 | 0.545-0.720 |  |  |  |
| Smoker (vs. non-smoker) | 0.914 | 0.757-1.103 |  |  |  |
| Systolic BP (mmHg) | 1.024 | 1.017-1.030 |  | 0.995 | 0.988-1.002 |
| Diastolic BP (mmHg) | 1.027 | 1.018-1.035 |  | 1.014 | 1.005-1.024 |
| Body mass index (kg/m^2^) | 1.047 | 1.025-1.070 |  | 1.027 | 1.000-1.056 |
| Waist circumference (cm) | 1.026 | 1.018-1.034 |  | 1.004 | 0.994-1.014 |
| Hemoglobin (g/dL) | 1.135 | 1.085-1.188 |  | 1.218 | 1.119-1.326 |
| Fasting glucose (mg/dL) | 1.031 | 1.022-1.039 |  | 1.000 | 0.991-1.010 |
| Hemoglobin A1c (%) | 3.270 | 2.578-4.149 |  | 1.123 | 0.868-1.453 |
| Triglycerides (mg/dL) | 1.001 | 1.000-1.001 |  |  |  |
| HDL-cholesterol (mg/dL) | 0.984 | 0.977-0.990 |  | 0.997 | 0.990-1.004 |
| LDL-cholesterol (mg/dL) | 1.007 | 1.001-1.013 |  | 1.008 | 1.002-1.015 |
| CMI | 1.052 | 1.009-1.097 |  | 0.942 | 0.891-0.995 |
| 25-Vitamin D (ng/mL) | 1.059 | 1.039-1.080 |  | 1.027 | 1.006-1.049 |
| UACR (mg/g creatinine) | 1.001 | 0.997-1.005 |  |  |  |

*****defined as eGFR between 60 and 89 mmHg.

Model I, adjusted for age and sex.
